# Relationship between personality, adherence to (mental) health behaviours and psychological distress during the COVID-19 pandemic

**DOI:** 10.1192/j.eurpsy.2022.1270

**Published:** 2022-09-01

**Authors:** A.T. Pereira, C. Cabacos, S. Soares, A. Araújo, A. Manão, A.P. Amaral, R. Sousa, A. Macedo

**Affiliations:** 1 Faculty of Medicine of University of Coimbra, Institute Of Psychological Medicine, Coimbra, Portugal; 2 Coimbra Institute for Biomedical Imaging and Translational Research, -, Coimbra, Portugal; 3 Centro Hospitalar e Universitário de Coimbra, Department Of Psychiatry, Coimbra, Portugal; 4 Centro Hospitalar e Universitário de Coimbra, Coimbra, Portugal, Department Of Psychiatry, Coimbra, Portugal; 5 Institute Polytechnic, Coimbra Health School, Coimbra, Portugal; 6 Fernão de Magalhães Health Center, Ars Centro, Coimbra, Portugal

**Keywords:** Covid-19, Adherence (to the Recommendations for Mental Health, personality

## Abstract

**Introduction:**

Public health authorities around the world have been disseminating messages to support mental health and psychosocial well-being during the COVID-19 pandemic. Based on the Portuguese guidelines, we have developed the Adherence Scale to the Recommendations for Mental Health during the COVID-19 Pandemic (ASR-MH-COVID19) to better understand this health behaviour.

**Objectives:**

To analyse the relationship between sociodemographics, personality traits, Adherence (to the Recommendations for Mental Health during the COVID-19 Pandemic) and psychological distress.

**Methods:**

413 individuals (69.2% female; mean age=31.02±14,272) completed an on-line survey, in September-December 2020, including sociodemographic questions, ASR-MH-COVID19, NEO-FFI-20 and Depression Anxiety Stress Scale (DASS-21) and Health Perception Scale.

**Results:**

Adherence scores did not significantly differ by gender, age and years of education. Women presented higher DASS and Neuroticism scores (p<.01). Adherence were negatively correlated with Neuroticism (r=-.247) and with Depression/Anxiety/Stress (all r».-200), positively with Openness to Experience (r=.174), Conscientiousness (r=.194) and Perceived Health (Physical, r=.173 and Psychological, r=.215) (all p<.01). Mediation analysis (Hays’ Macro Process - Model 4) revealed that Adherence is a partial mediator between Openness and DASS and Conscientiousness and DASS; when considering Neuroticism, only the direct effect was significant. The effect of Perceived Health (both Physical and Psychological) on DASS was also mediated by Adherence.

**Conclusions:**

The Health Behaviour Model proposes a pathway linking personality and health that applies to these results about adherence and psychological distress during the COVID-19 pandemic. Personality and perceived health (also a trait) influence both adherence to mental health behaviours and psychological distress. Understanding personality is vital for health care providers.

**Disclosure:**

No significant relationships.

